# Supplementary material for: miR-301a Suppression within Fibroblasts Limits the Progression of Fibrosis through the TSC1/mTOR Pathway
Source: Mol Ther Nucleic Acids. 2020 May 26;21:217–28. doi: 10.1016/j.omtn.2020.05.027 (PMC7321782; doi:10.1016/j.omtn.2020.05.027)
Supplement: Document S1. Figures S1–S5 and Table S1 [file mmc1.pdf]

OMTN, Volume *21*

Supplemental Information

miR-301a Suppression within Fibroblasts Limits the Progression of Fibrosis through the TSC1/mTOR Pathway

Jiexuan Wang, Xun Li, Mingtian Zhong, Yansheng Wang, Liming Zou, Miaomiao Wang, Xiaoli Gong, Xinjie Wang, Chengzhi Zhou, Xiaodong Ma, and Ming Liu

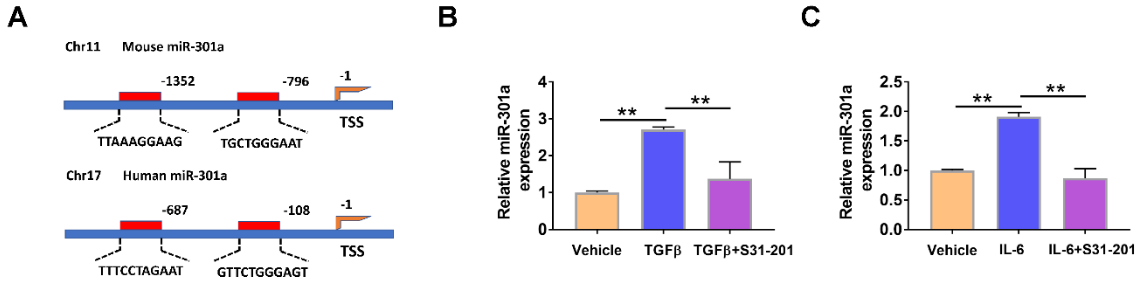

Supplementary Figure 1. miR-301a expression in fibroblast activation is dependent on Stat3. **(A)** Online prediction indicated that transcription factor Stat3 might bind to the miR-301a promoter in both mouse and human. Fibroblasts were treated with TGF-β **(B)** or IL-6 **(C)** and Stat3 inhibitor, S31-201 (30μM) at 24 h and the expression of miR-301a was determined by qPCR. Values are means ± sd. ***P* ≤0.01 indicate the significance of the differences between the indicated groups.

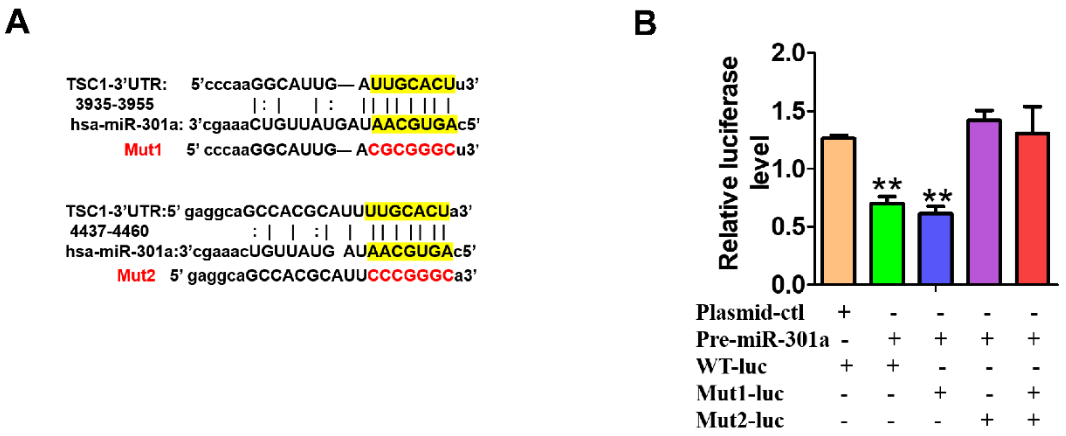

Supplementary Figure 2. The true binding site of miR-301a with TSC1. (A) Prediction of major interference sites between miR-301a and TSC1 mRNA 3'UTR by using Targetscan. Mutant 3′UTR contains mutations that abolished the seed match with miR-301a. **(B)** Luciferase activity in 293T cells transfected with the indicated luciferase reporters.

A

miR-130a CAGUGCAAUGUUAAAAGGGCAU
miR-130b CAGUGCAAUGAUGAAAGGGCAU
miR-301a CAGUGCAAUAGUAUUGUCAAAGC
miR-301b CAGUGCAAUGAUAUUGUCAAAGC

B

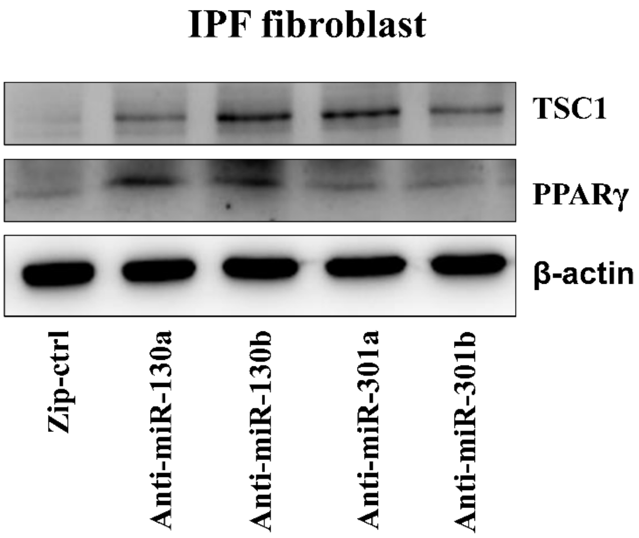

Supplementary Figure 3. (A) Prediction of major interference sites between miR-301a and TSC1 mRNA 3’UTR by using Targetscan. **(B)** Luciferase activity in 293T cells transfected with the indicated luciferase reporter with either a control plasmid or precursor miR-301a plasmid.

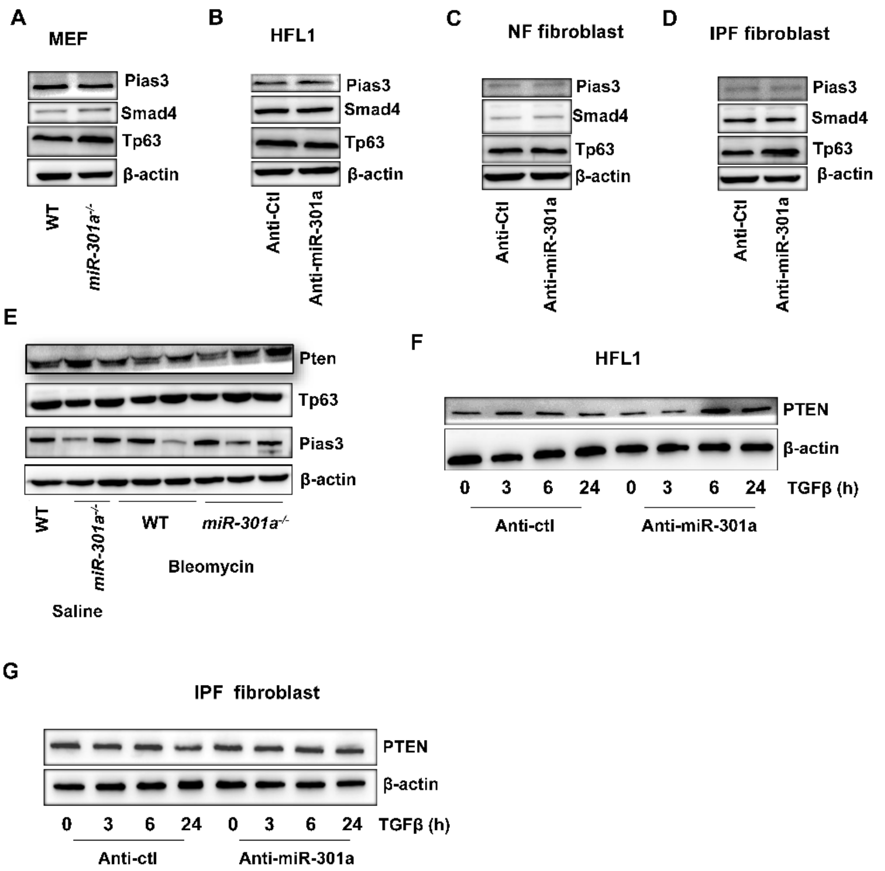

Supplementary Figure 4. Expression of miR-301a targets in fibroblast cells. (A) Western blot analysis of the expression of miR-301a targets Pias3, Smad4, and Tp63 in WT and *miR-301a*$^{-/-}$ MEFs. **(B–D)** Western blot analysis of the expression of miR-301a targets Pias3, Smad4, and Tp63 in HFL1 cells **(B)**, NF fibroblasts isolated from a normal donor **(C)**, and fibroblasts from patients with IPF **(D)**, which were transfected with either Anti-Ctl or Anti-miR-301a. **(E)** Western blot analysis of the expression of miR-301a targets PTEN, Tp63, and Pias3 in lung tissues from WT and *miR-301a*$^{-/-}$ mice with the indicated treatment. Western blot analysis of PTEN expression in HFL1 cells **(F)** and IPF fibroblasts **(G)**, which were transfected with either Anti-Ctl or Anti-miR-301a.

A

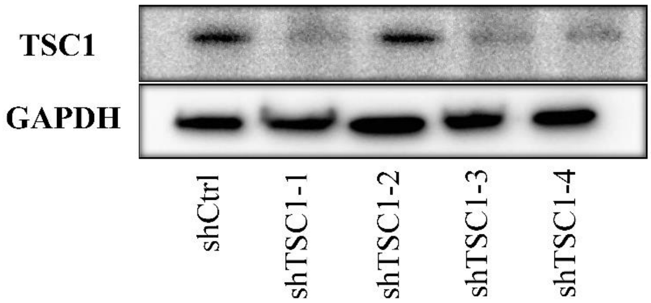

Supplementary Figure 5. Western blot shows the efficacy of shRNA knockdown of TSC1 in 293T cells.

Supplementary Table 1. The characteristics of IPF patient.

Characteristics	Range（N=8）
Age	54.75（36-64）
Sex	
Male	3
Female	5
Pathologic diagnosis	UIP
Therapy	
Prednisone	8
N-Acetyl-L-cysteine	8
Nintedanib	2
Cyclophosphamide(CTX)	1
Azathioprine	1
