# Supplementary material for: miR-301a Suppression within Fibroblasts Limits the Progression of Fibrosis through the TSC1/mTOR Pathway
Source: Mol Ther Nucleic Acids. 2020 May 26;21:217–28. doi: 10.1016/j.omtn.2020.05.027 (PMC7321782; doi:10.1016/j.omtn.2020.05.027)
Supplement: Document S2. Article plus Supplemental Information [file mmc2.pdf]

Molecular Therapy
Nucleic Acids
Original Article

# miR-301a Suppression within Fibroblasts Limits the Progression of Fibrosis through the TSC1/mTOR Pathway

Jiexuan Wang,[1,3] Xun Li,[2,3] Mingtian Zhong,[2,3] Yansheng Wang,[1] Liming Zou,[2] Miaomiao Wang,[2] Xiaoli Gong,[2] Xinjie Wang,[2] Chengzhi Zhou,[1] Xiaodong Ma,[2] and Ming Liu[1]

[1]National Clinical Research Center for Respiratory Disease, State Key Laboratory of Respiratory Disease, Guangzhou Institute of Respiratory Health, The First Affiliated Hospital of Guangzhou Medical University, Guangzhou 510120, China; [2]Institute for Brain Research and Rehabilitation, Guangdong Key Laboratory of Mental Health and Cognitive Science, Center for Studies of Psychological Application, South China Normal University, Guangzhou 510631, China

**Pulmonary fibrosis has been characterized by abnormal proliferation of fibroblasts and massive deposition of the extracellular matrix, which results from a complex interplay of chronic injury and inflammatory responses. MicroRNA-301a (miR-301a) is activated by multiple inflammatory stimulators, contributing to multiple tumorigenesis and autoimmune diseases. This study showed that miR-301a was overexpressed in a bleomycin-induced murine model of pulmonary fibrosis and patients with idiopathic pulmonary fibrosis (IPF). In addition, miR-301a was activated by transforming growth factor β (TGF-β) and interleukin 6 (IL-6) in normal and IPF fibroblasts, which was markedly reversed by the signal transducer and activator of transcription 3 (STAT3) inhibitor. The genetic ablation of miR-301a in mice reduced bleomycin-induced lung fibrosis, and the downregulation of miR-301a restrained proliferation and activation of fibroblasts. Furthermore, this study demonstrated that TSC1 was a functional target of miR-301a in fibroblasts, and the negative regulation of TSC1 by miR-301a promoted the severity of pulmonary fibrosis through the mammalian target of rapamycin (mTOR) signaling pathway. The blocking of miR-301a by the intravenous injection of antagomiR-301a inhibited the proliferation of fibroblasts and the structural destruction of lung tissues in the bleomycin-induced lung fibrosis mouse model. The findings revealed the crucial role of the miR-301a/TSC1/mTOR axis in the pathogenesis of pulmonary fibrosis, suggesting that miR-301a might serve as a potential therapeutic target.**

## INTRODUCTION

Pulmonary fibrosis is characterized by lung epithelial cell damage, proliferation of fibroblasts, and aggregation of extracellular matrix, with inflammatory injury and the epithelial-mesenchymal transition (EMT), ultimately leading to the structural destruction of the lung tissue (the end-stage change of many lung diseases).[1,2] The most common disease characterized by pulmonary fibrotic lesions is idiopathic pulmonary fibrosis (IPF). The average survival after diagnosis of IPF was only 2.8 years, and the 5-year survival rate was less than 30%.[2] In 2014, the US Food and Drug Administration approved two drugs, nintedanib and pirfenidone, for the treatment of pulmonary fibrosis; however, still no drugs are available to improve patient survival.[1,3,4] Many studies have shown that pulmonary fibrosis was closely related to chronic inflammation and angiogenesis. Several cytokines, such as transforming growth factor β (TGF-β), interleukin 1β (IL-1β), IL-6, and tumor necrosis factor α (TNF-α), are demonstrated to be the main drivers of fibrosis.[5] Therefore, attempts should be made to explore the pathogenesis of fibrosis and inflammation-driven signaling pathways and find new targets for treating fibrosis.

MicroRNAs (miRNAs) are small (20–24 nt) noncoding RNAs involved in the post-transcriptional regulation of gene expression in multicellular organisms by affecting both the stability and translation of mRNAs.[6] The miR-130/301 family includes miR-130a, miR-130b, miR-301a, miR-301b, and miR-454, which share a common seed sequence and target a common sequence.[7] All of these miRNAs have been demonstrated to be activated by the YAP/TAZ transcription factors to promote the progression of fibrosis in mice with lung and liver fibrosis.[8] In particular, miR-130 was co-localized with vimentin and α-smooth muscle actin (α-SMA) and found to have a positive correlation with YAP nuclear localization. In addition, miR-130 directly targeted peroxisome proliferator-activated receptor

Received 14 April 2020; accepted 22 May 2020;
https://doi.org/10.1016/j.omtn.2020.05.027.

[3]These authors contributed equally to this work.

**Correspondence:** Xiaodong Ma, Institute for Brain Research and Rehabilitation, South China Normal University, Guangzhou 510006, China.
**E-mail:** sciencema@hotmail.com
**Correspondence:** Chengzhi Zhou, National Clinical Research Center for Respiratory Disease, State Key Laboratory of Respiratory Disease, Guangzhou Institute of Respiratory Health, The First Affiliated Hospital of Guangzhou Medical University, Guangzhou 510120, China.
**E-mail:** doctorzcz@163.com
**Correspondence:** Ming Liu, National Clinical Research Center for Respiratory Disease, State Key Laboratory of Respiratory Disease, Guangzhou Institute of Respiratory Health, The First Affiliated Hospital of Guangzhou Medical University, Guangzhou 510120, China.
**E-mail:** mingliu128@hotmail.com


This is an open access article under the CC BY license (http://creativecommons.org/licenses/by/4.0/).

γ (PPARγ) and endothelin-1 in pulmonary hypertension vascular cells.[8] A network-based bioinformatics approach demonstrated that the miR-130/301 family affected the pulmonary arterial endothelial cells and smooth muscle cells in pulmonary hypertension.[8] This miRNA family did not exert similar control in both endothelial cells and smooth muscle cells. For example, miR-130/301 controlled pulmonary smooth muscle cells via the regulation of miR-204 and signal transducer and activator of transcription 3 (STAT3), but controlled the proliferation of pulmonary endothelial cells and apoptotic signaling by regulating apelin, miR-424/503, and FGF2. These findings suggested the complex regulatory networks of this miRNA family. The true influence of individual miRNAs in fibrotic pathways may be more widespread and hence needs to be explored.

Tuberous sclerosis complex is an autosomal-dominant disorder affecting various organ systems. It results from mutations in TSC1 (hamartin) and TSC2 (tuberin).[9] TSC1 and TSC2 form a complex that negatively regulates the activity of the mammalian target of rapamycin (mTOR) by inhibiting Ras homolog enriched in brain (Rheb).[10] mTOR is a serine/threonine kinase that plays an important role in cell proliferation, growth, and survival. mTOR interacts with several proteins to form two distinct complexes: mTOR1 and mTOR2. mTOR1 controls cell growth and metabolism, while mTOR2 is involved in cell survival.[11–13] The active form of Rheb enhances the phosphorylation of mTOR substrates p70S6 kinase (S6K) and 4E-BP1, and it promotes cell growth and proliferation.[9] The phosphatidylinositol 3-kinase (PI3K)/mTORC1/4E-BP1 axis affects differentiation, collagen production, and proliferation of myofibroblasts induced by TGF-β.[14,15] Recently, the overexpression of TSC1 and TSC2 was found to decrease the proliferation and differentiation of myofibroblasts.[16] These observations indicated the critical role of TSC1/2 and mTOR in regulating human fibrotic phenotypes.

The present study showed that miR-301a was activated by TGF-β and IL-6 stimulation in fibroblasts and overexpressed in a murine model of fibrosis and patients with IPF. The genetic ablation of miR-301a in mice protected against bleomycin-induced lung fibrosis. In addition, the upregulation of TSC1 and the deactivation of mTOR in fibroblasts with miR-301a ablation coincided with the reduced severity of fibrotic tissue destruction in the lungs. The results demonstrated that the miR-301a/TSC1/mTOR axis was a key positive regulator of fibrogenesis, providing a new target for the clinical treatment of pulmonary fibrosis.

## RESULTS

### Overexpression of miR-301a in Fibroblasts Depended on STAT3 Levels

To evaluate the role of miR-301a in pulmonary fibrosis, the expression of miR-301a was measured first in patients with IPF. The expression levels of miR-301a mRNA were significantly higher in lung tissues from patients with IPF than from normal donors. This result was further demonstrated in fibroblasts isolated from the lung tissues of patients with IPF and from the nonfibrotic lung tissues of normal donors (Figures 1A and 1B). The intratracheal instillation of bleomycin-induced pulmonary fibrosis mouse models was also used to evaluate whether the expression level of miR-301a was higher in mice with fibrosis. The results showed that the ΔCt values of miR-301a normalized to a reference RNA (sno292) were significantly lower (Figure 1C) in lung tissues from mice 1 week after bleomycin instillation compared with saline-treated controls. The difference in ΔCt values represented a >32-fold upregulation of miR-301a. Correspondingly, 2 and 3 weeks after treatment, the expression levels of miR-301a were higher compared with the levels after saline treatment (Figure 1C).

TGF-β and IL-6 are the two main cytokines involved in fibroblast proliferation and activation. The mouse embryonic fibroblasts (MEFs), normal fibroblasts (HFL1), and fibroblasts isolated from patients with IPF (IPF fibroblasts) were treated with TGF-β and IL-6 for 12 and 24 h, and the expression levels of miR-301a were determined. As expected, the expression levels of miR-301a significantly increased after TGF-β (Figures 1D–1F) or IL-6 (Figures 1G–1I) treatment. Moreover, bioinformatics analysis indicated that STAT3 might bind to the miR-301a promoter (Figure S1A). Both TGF-β and IL-6 induced STAT3 activation. Then, whether the two cytokines mediated miR-301a upregulation via inducing STAT3 activation in fibroblasts was determined. The effect of miR-301a induction was found to be abrogated with the treatment of STAT3 inhibitor S31-201 (Figures S1B and S1C).

### Response of Wild-Type (WT) and *miR301a*$^{-/-}$ Mice to Bleomycin

Bleomycin has been widely used in rodent pulmonary fibrosis models to study the mechanism of fibrosis. In this study, bleomycin or normal saline (control group) was intratracheally instilled in WT and *miR-301a*$^{-/-}$ mice, and lung tissues were collected on days 14 and 21 after bleomycin or normal saline treatment (Figure 2A). First, the severity of fibrosis was evaluated in WT and *miR-301a*$^{-/-}$ mice treated with bleomycin. Compared with WT controls, *miR-301a*$^{-/-}$ mice showed significantly less body weight loss (Figure 2B). Next, the survival of WT and *miR-301a*$^{-/-}$ mice after bleomycin administration was evaluated. WT mice and *miR-301a*$^{-/-}$ mice began to die on day 4 and day 12, respectively, after bleomycin treatment. *miR-301a*$^{-/-}$ mice showed significantly increased survival rates compared with their WT littermates (Figure 2C). No mice treated with normal saline died. Next, the changes in lung histology were evaluated 21 days after the bleomycin injection. In WT mice, extensive fibrosis and collagen deposition in lung tissues were found on day 21. In contrast, *miR-301a*$^{-/-}$ mice had dramatically less pulmonary fibrosis on day 21 (Figures 2D–2F). Furthermore, the expression levels of α-SMA, which is a marker of smooth muscle cells and is currently considered a marker of myofibroblasts,[17] were markedly reduced in *miR-301a*$^{-/-}$ mice (Figure 2G). Accordingly, the expression levels of fibronectin (Fn), vimentin, and α-SMA, which are markers of fibrosis, were all significantly lower in *miR-301a*$^{-/-}$ mice than in WT mice, as demonstrated by western blot analysis (Figure 2H). These observations indicated that the genetic deletion of miR-301a reduced the severity of lung fibrosis following bleomycin injection.

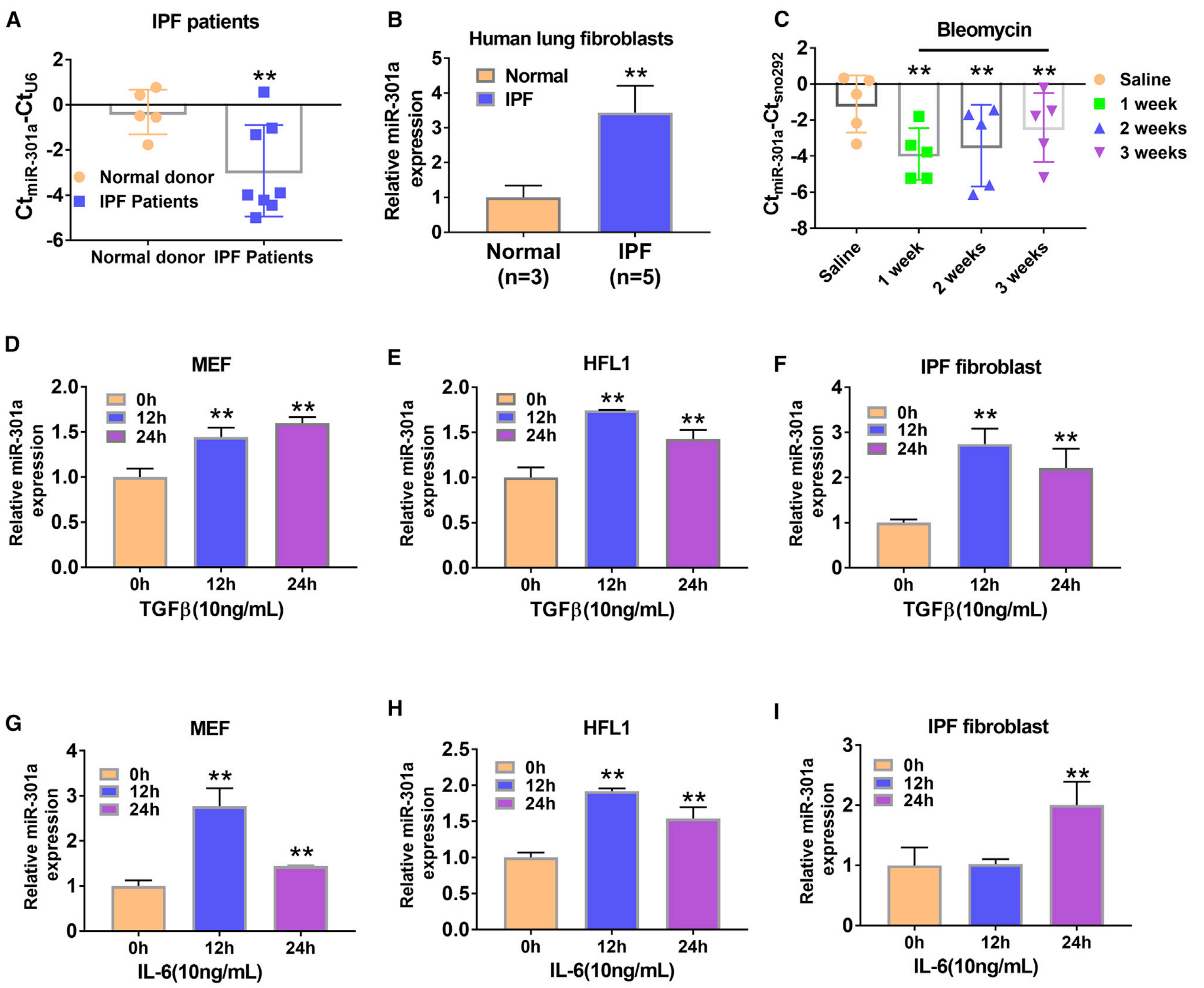


**Figure 1. Inflammation-Induced miR-301a Upregulation in Fibrotic Fibroblasts**

(A) The expression of miR-301a in lung tissues from patients with idiopathic pulmonary fibrosis (IPF) and normal donors, as determined by qPCR. (B) The expression of miR-301a in fibroblasts isolated from the lungs of patients with IPF lung and from nonfibrotic lungs of normal donors, as determined by qPCR. (C) The expression of miR-301a during bleomycin-induced lung fibrosis. Lung tissue was harvested by tracheal infusion in bleomycin-induced WT mice (n = 5, each group) during and 1 week, 2 weeks, and 3 weeks after pulmonary fibrosis. The expression of miR-301a was detected by qPCR, and an equal dose of saline was perfused into the control group. (D–F) miR-301a expression was induced by TGF-β (10 ng/mL) stimulation of mouse embryonic fibroblasts (MEFs, D), HFL1 cells (E), and IPF fibroblasts isolated from patients (F). (G–I) miR-301a expression was induced by IL-6 stimulation of MEFs (G), HFL1 cells (H), and IPF fibroblasts isolated from patients (I). Values are expressed as mean ± standard deviation. **$p \leq 0.01$, for differences between the groups with and without treatment or between the indicated groups.

### miR-301a Regulated TGF-β- and IL-6-Induced Fibroblast Activation

Fibrosis is characterized by the accumulation of myofibroblasts, which are derived from activated fibroblasts. Several cytokines, such as TGF-β and IL-6, have been reported to promote fibroblast proliferation and differentiation. Hence, this study explored whether miR-301a inhibition reduced fibroblast proliferation. Introducing the miR-301a inhibitor locked nucleic acid (LNA)-anti-miR-301a led to the inhibition of proliferation of HFL1 cells and IPF fibroblasts. Upon TGF-β stimulation, miR-301a inhibition with LNA-anti-miR-301a significantly reduced the proliferation of HFL1 cells and IPF fibroblasts (Figures 3A and 3B). Treatment with TFG-β elicited an increment in the expression levels of Fn, vimentin, and α-SMA in both HFL1 cells and IPF fibroblasts, which were significantly reduced when LNA-anti-miR-301a was introduced into HFL1 cells and IPF fibroblasts (Figures 3C and 3D). As miR-301a was demonstrated to be an activator of STAT3 in various cells,[18,19] whether miR-301a inhibition reduced the activation of STAT3 was investigated next. As expected, less STAT3 activation was found after miR-301a inhibition in TGF-β-treated IPF fibroblasts (Figures 3E and 3F). These data suggested that miR-301a was required

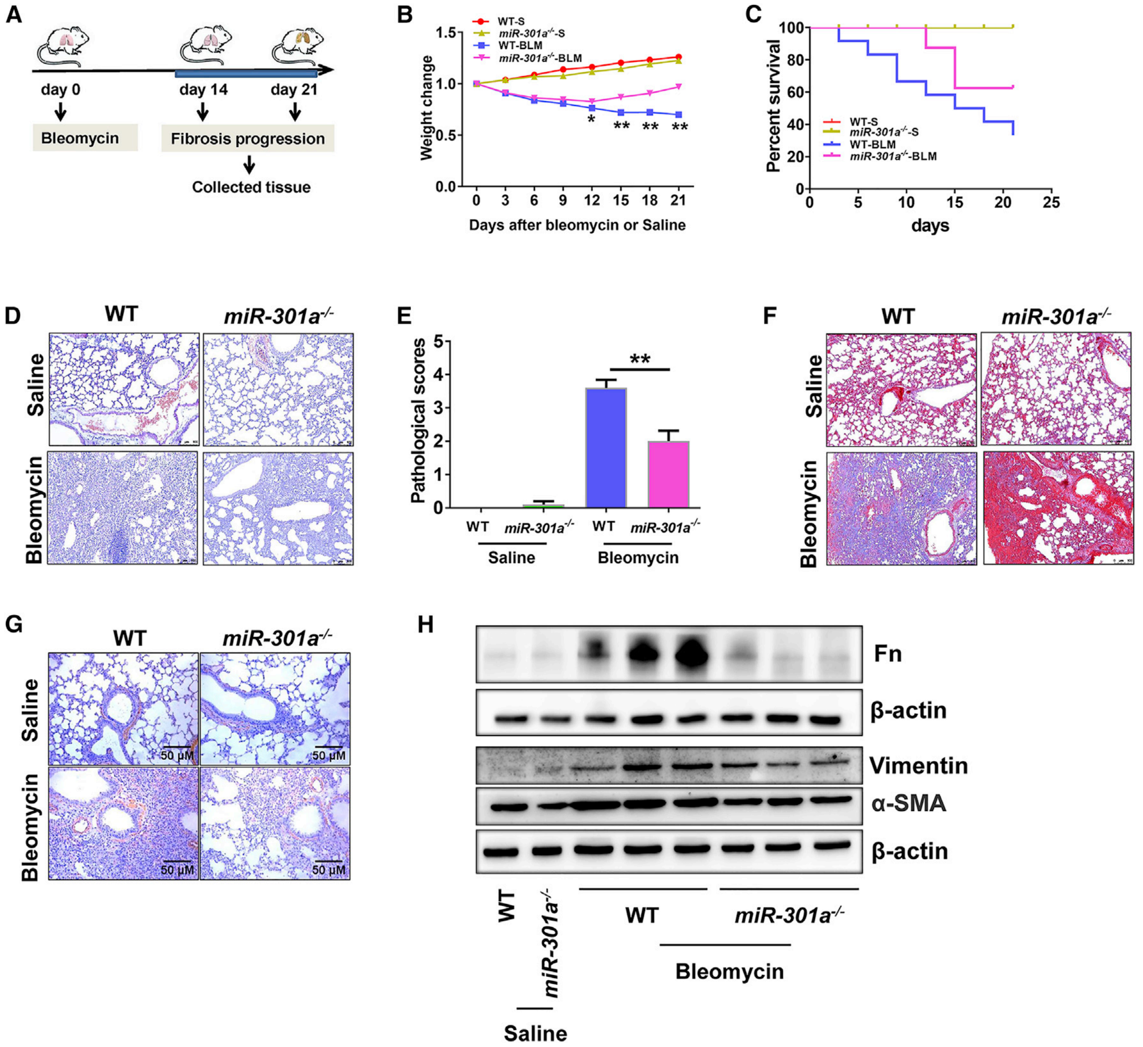


**Figure 2. Response of WT and *miR-301a*$^{-/-}$ Mice to Bleomycin**

(A) Schematic representation of the intratracheal instillation of bleomycin or saline into WT and *miR-301a*$^{-/-}$ mice, respectively (saline, n = 15; bleomycin, n = 20). (B) Body weight loss during bleomycin-induced lung fibrosis in WT (n = 5) and *miR-301a*$^{-/-}$ (n = 5) mice. (C) Survival of bleomycin-treated WT (n = 10) and *miR-301a*$^{-/-}$ mice (n = 15). The survival of the mice was monitored daily. The analysis group was found to be significantly different using the log rank test ($p < 0.0001$). (D) Hematoxylin and eosin (H&E) histological sections of lung tissues after the indicated treatment. Scale bars, 100 μm. (E) Pathological scoring of lung tissues from (D). (F) Masson's trichrome staining showed a significant increase in fibrosis in lung tissues isolated after the indicated treatment. Scale bars, 100 μm. (G) Immunohistochemical analysis of α-SMA in lung tissues isolated after the indicated treatment. Scale bars, 50 μm. (H) The expression of fibronectin (Fn), vimentin, and α-SMA in lung tissues from WT (n = 5) and *miR-301a*$^{-/-}$ (n = 5) mice treated with saline or bleomycin was detected by western blot analysis. Values are expressed as mean ± standard deviation. *$p < 0.05$, **$p < 0.01$, for differences between the groups with and without treatment or between the indicated groups.

for not only proliferation of fibroblasts but also response of fibroblasts to cytokines.

### miR-301a Directly Targeted TSC1 in Fibroblasts

A previous study analyzed RNA22, miRWalk, miRanda, TargetScan, PITA, and miRDB databases to construct networks generated using miR-301a-predicted target genes.[20] Cell proliferation was the main biological signaling pathway. From the list of target genes of miR-301a, the present study focused on TSC1 and PTEN due to their anti-proliferative function. Thus, the study verified whether TSC1 was a direct target gene of miR-301a. The miRNA-target prediction analysis revealed a major binding site for miR-301a within the TSC1 RNA 3′ UTR (Figure 4A). A luciferase reporter assay showed that miR-301a directly bound to TSC1 mRNA and downregulated TSC1 expression in 293T cells (Figures 4B and 4C). The true binding site of miR-301a with the TSC1 3′ UTR was further verified (Figures S2A and 2B). Next, MEFs were isolated from WT and *miR-301a*$^{-/-}$ mice to evaluate PTEN and TSC1 expression. TSC1 expression was significantly upregulated, whereas PTEN expression was not altered in *miR-301a*$^{-/-}$ MEFs

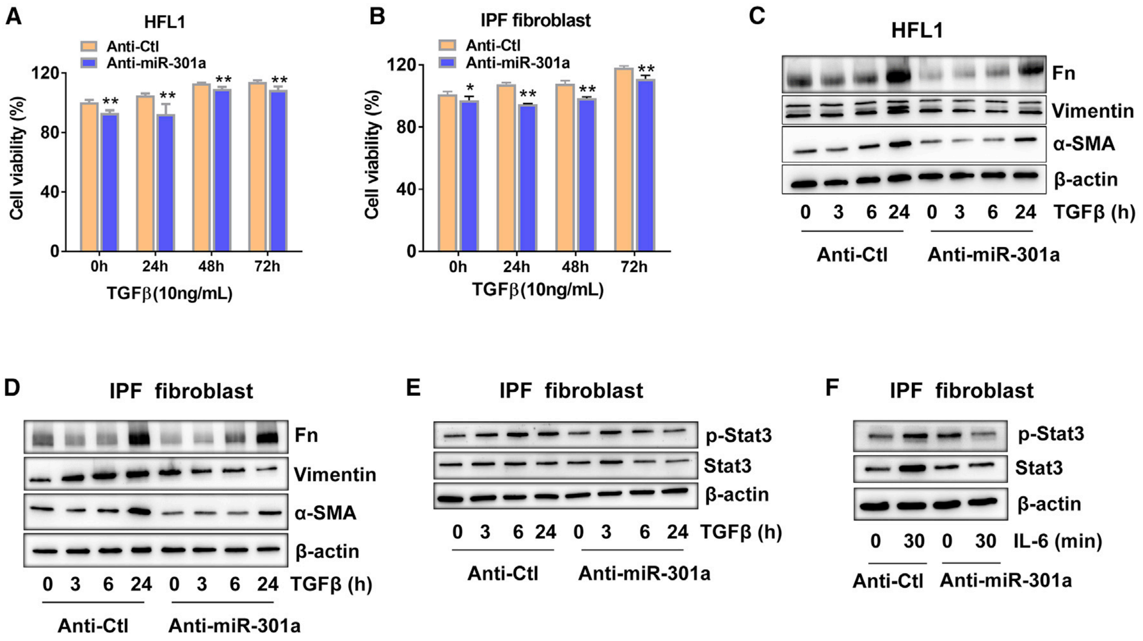


**Figure 3. miR-301a Regulated TGF-β-Induced Fibroblast Activation**

(A and B) Lung fibroblasts, HFL1 cells (A), and lung fibroblasts (B) isolated from patients with IPF (IPF fibroblasts) were transfected with LNA-anti-control (anti-Ctl) or LNA-anti-miR-301a (anti-miR-301a) for 48 h and treated with TGF-β (10 ng/mL) for 24, 48, and 72 h, respectively. Cell proliferation was measured using a CCK-8 assay. (C and D) Lung fibroblasts, HFL1 cells (C), and IPF fibroblasts (D) were transfected with anti-Ctl or anti-miR-301a for 48 h and treated with TGF-β (10 ng/mL) for 3, 6, and 24 h, respectively. The expression of Fn, vimentin, and α-SMA was measured using western blot analysis. (E and F) IPF fibroblasts were transfected with anti-Ctl or anti-miR-301a for 48 h and treated with TGF-β (10 ng/mL) (E) or IL-6 (10 ng/mL) (F) at the indicated times. The expression of phosphorylated Stat3 (p-Stat3) and Stat3 was measured by western blot analysis. Values are expressed as mean ± standard deviation. $^{*}p < 0.05$, $^{**}p < 0.01$, for differences between anti-Ctl and anti-miR-301a groups.

compared with WT MEFs (Figure 4D). In HFL1 cells, TSC1 expression was also significantly upregulated in cells transfected with LNA-anti-miR-301a compared with cells transfected with LNA-anti-control (Figure 4E). In addition, fibroblasts were isolated from three patients with IPF and reduced miR-301a expression by introducing LNA-anti-miR-301a. As shown in Figure 4F, miR-301a inhibition with 50 pmol of LNA-anti-miR-301a significantly upregulated the expression of TSC1 in fibroblasts from the three patients. Furthermore, TSC1 expression was upregulated in fibroblasts from IPF patients by introducing the inhibitor of other miR-130/301 family members, including miR-130a, miR-130b, and miR-301b. However, upregulation of PPARγ was only found in fibroblasts with the inhibitors of miR-130a or miR-130b (Figures S3A and S3B). Importantly, the expression levels of TSC1 were significantly lower in lung tissues or fibroblasts from patients with IPF than from normal donors (Figures 4G and 4H). Taken together, these data indicated that miR-301a directly targeted TSC1 mRNA in fibroblasts.

### Inhibition of Lung Fibrosis in *miR-301a*$^{-/-}$ Mice Correlated with the Deactivation of STAT3 and mTOR through the miR-301a Target in Fibroblasts

Cell proliferation in fibrotic lung tissues was measured to evaluate the role of miR-301a and TSC1 in regulating lung fibrosis. Significantly fewer Ki67$^{+}$ cells were found in *miR-301a*$^{-/-}$ mice compared with WT mice treated with bleomycin (Figures 5A and 5B). Previous studies demonstrated that TSC1 was a direct target of miR-301a. Therefore, the expression of TSC1 in fibrotic lung tissues was evaluated in WT and *miR-301a*$^{-/-}$ mice. As shown in Figures 5C and 5D, the expression level of TSC1 was significantly higher in *miR-301a*$^{-/-}$ mice than in WT mice. The present study also probed the expression of other miR-301a target genes (PTEN, PIAS3, TP63, and SMAD4) in either fibroblasts or fibrotic lung tissues in mice. However, the expression levels of PTEN, Pias3, Tp63, and SMAD4 remained largely unchanged (Figures S4A–S4E). In particular, the expression levels of PTEN were measured in both fibrotic mice and fibroblasts because PTEN was demonstrated to be an miR-301a target gene in multiple cells. The pathway enrichment of miR-130/301 targets revealed that PTEN was involved in multiple signaling pathways in pulmonary hypertension.[8] Although the knockdown of miR-301a via LNA-anti-miR-301a significantly enhanced PTEN expression in TGF-β-treated HFL1 cells, PTEN expression was not altered in IPF fibroblasts and lung tissues from fibrotic mice (Figures S4F and S4G). Collectively, these data demonstrated that miR-301a-mediated target suppression might be effective for TSC1 but not all miR-301a gene targets in the fibrotic lungs.

Next, mTOR activation in lung tissues and three types of fibroblasts (MEFs, HFL1 cells, and IPF fibroblasts) was measured. As expected, significantly less mTOR and phosphorylated (p-)P70S6K1 activation

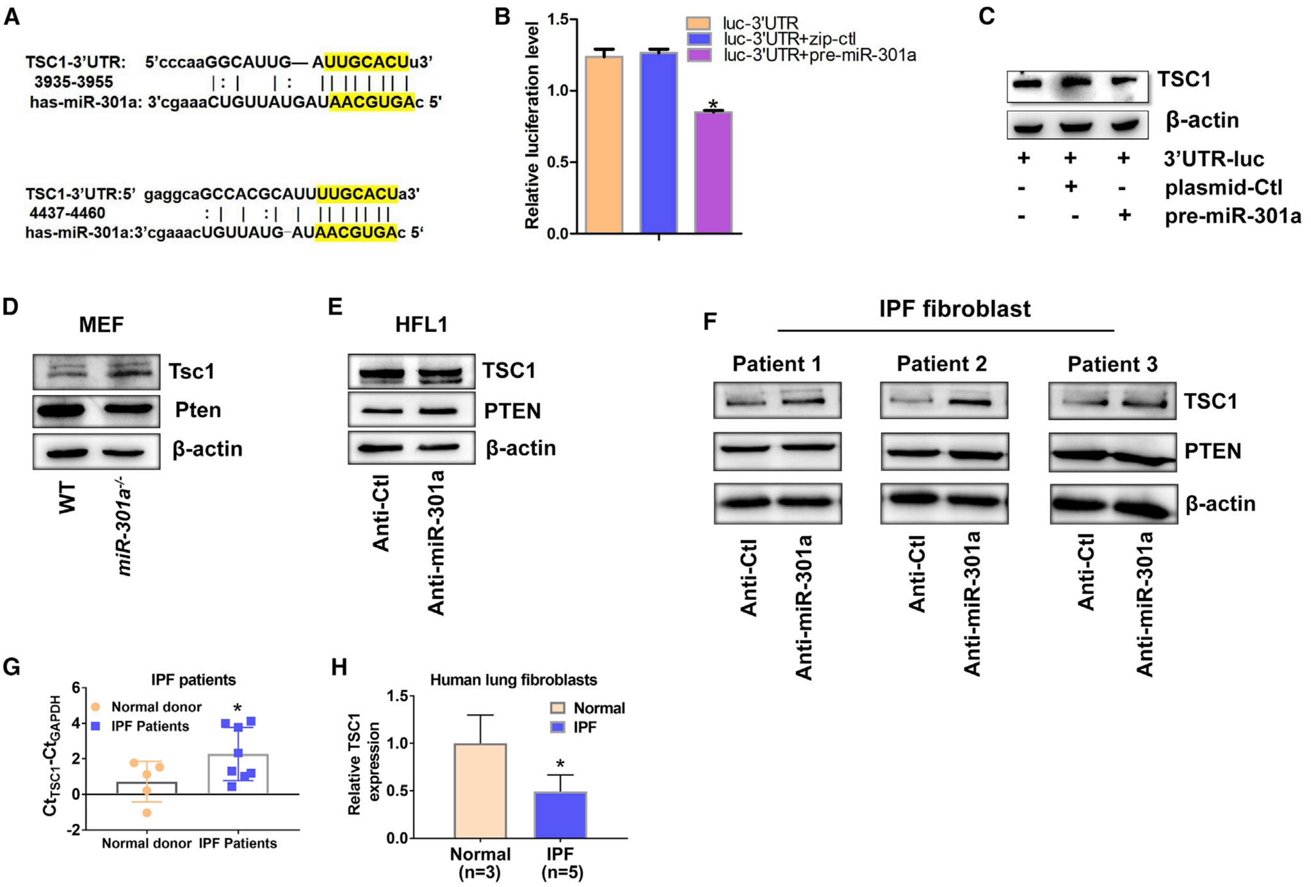


**Figure 4. miR-301a Directly Targeted TSC1 in Fibroblasts**

(A) Prediction of major interference sites between miR-301a and the TSC1 mRNA 3′ UTR using TargetScan. (B) Luciferase activity in 293T cells transfected with the indicated luciferase reporter with either a control plasmid or a precursor miR-301a plasmid. (C) Western blot analysis of TSC1 expression in 293T cells with the indicated luciferase reporter with either a control plasmid or a precursor miR-301a plasmid. (D) Western blot analysis of Tsc1 and PTEN expression in MEFs isolated from WT or *miR-301a*$^{-/-}$ mice. (E) Western blot analysis of TSC1 and PTEN expression in HFL1 cells transfected with either anti-Ctl or anti-miR-301a. (F) Western blot analysis of TSC1 and PTEN expression in IPF fibroblasts transfected with either anti-Ctl or anti-miR-301a. (G) The expression of TSC1 in lung tissues from patients with IPF and normal donors, as determined by qPCR. (H) The expression of TSC1 in fibroblasts isolated from the lungs of patients with IPF lung and from nonfibrotic lungs of normal donors, as determined by qPCR. Values are expressed as mean ± standard deviation. *$p < 0.05$, for differences between the indicated groups.

was found in the lung tissues of *miR-301a*$^{-/-}$ mice compared with the lung tissues of WT mice (Figure 5E). Accordingly, mTOR and p-P70S6K1 showed remarkably lower expression levels in three different types of fibroblasts when miR-301a was inhibited (Figures 5F–5H). Consistent with previous reports, mTOR and p-P70S6K1 were constitutively activated in TGF-β-treated fibroblasts, as judged by the increased phosphorylation of mTOR and p-P70S6K1. However, significantly less mTOR and p-P70S6K1 activation and higher expression levels of TSC1 were found in fibroblasts with miR-301a inhibition compared with TGF-β-treated control fibroblasts (Figures 5I and 5J). Furthermore, the inhibition of miR-301a in IPF fibroblasts significantly reduced mTOR activation when the cells were treated with IL-6 (Figure 5K). Short hairpin RNA (shRNA)-TSC1 was introduced in IPF fibroblasts to further determine whether the reduction of mTOR with miR-301a inhibition was due to TSC1 expression. First, four shRNAs against TSC1 were used and shTSC1-1, shTSC1-3, and shTSC1-4 were found to significantly downregulate TSC1 expression in 293T cells (Figure S5A). Thus, shTSC1-1 was used for the following experiments. mTOR activation was significantly reduced when LNA-anti-miR-301a was introduced into IPF fibroblasts, whereas concomitant TSC1 knockdown reversed the status of mTOR activation (Figure 5L). Importantly, forced expression of TSC1 in fibroblast reversed the proliferative phenotype induced by miR-301a (Figure 5M). Collectively, the results suggested that miR-301a activated the mTOR pathway by negatively regulating TSC1 in fibroblasts and promoted the progression of pulmonary fibrosis.

### miR-301a Inhibition in WT Mice Ameliorated Pulmonary Fibrosis, which was Reversed by TSC1 Knockdown

Given the attenuation of pulmonary fibrosis in miR-301a-deficient mice, the present study investigated whether inhibiting miR-301a with an LNA-based oligonucleotide complementary to miR-301a

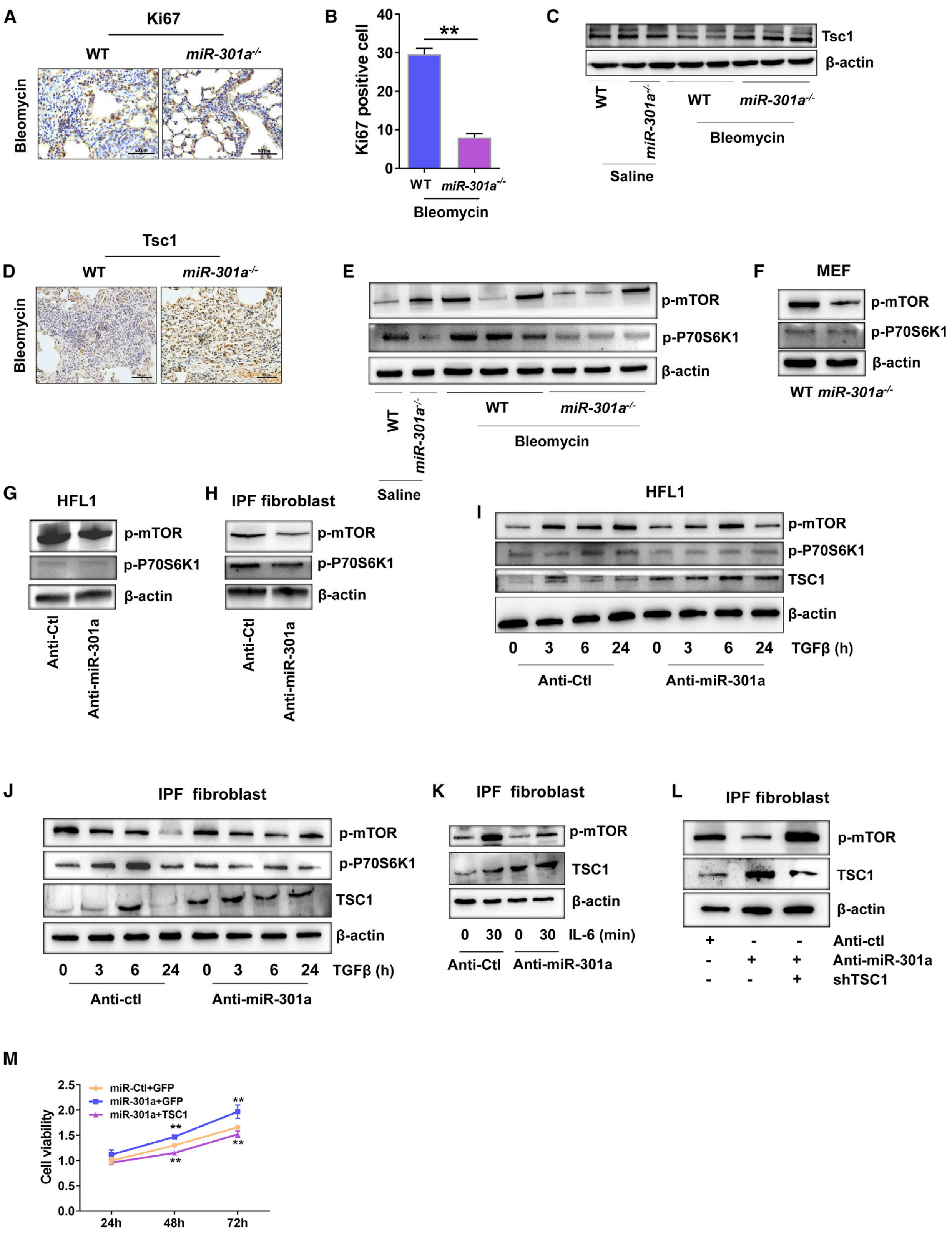


*(legend on next page)*

(anti-miR-301a) could effectively reduce bleomycin-induced pulmonary fibrosis. First, the anti-miR-301a was intravenously injected into WT mice for 5 consecutive days before bleomycin treatment (Figure 6A). Compared with anti-miRNA control, anti-miR-301a administration significantly downregulated miR-301a expression (Figure 6B) and upregulated its target TSC1 (Figure 6C). Consistent with gene expression, the severity of pulmonary fibrosis was consistently attenuated by anti-miR-301a (Figure 6D). Next, the study determined whether miR-301a inhibition reduced the injury in mice with fibrosis by elevating the expression levels of TSC1. The shTsc1 lentivirus was prepared against Tsc1 expression and intravenously injected into *miR-301a*$^{-/-}$ mice (Figure 6E). Compared with the control group, shTsc1 administration significantly downregulated TSC1 and upregulated its downstream genes p-mTOR (Figures 6F and 6G). The severity of pulmonary fibrosis significantly increased with shTsc1 administration in *miR-301a*$^{-/-}$ mice, as judged by hematoxylin and eosin (H&E) and α-SMA staining (Figure 6H). These observations complemented the findings of the present study that the inhibition of miR-301a and elevation of the expression levels of TSC1 in mice reduced pulmonary fibrosis, suggesting that prophylactic anti-miR-301a administration protected animals from bleomycin-induced pulmonary fibrosis.

## DISCUSSION

The present study provided a novel insight into the molecular mechanisms of pulmonary fibrosis. It found that the deletion of miR-301a alleviated pulmonary fibrosis by targeting TSC1, which negatively regulated its downstream mTOR pathway. Several fibrogenic growth factors and cytokines, such as TGF-β, platelet-derived growth factor (PDGF), IL-1β, IL-6, IFN-γ, and TNF-α, are considered to be the key regulators in the pathogenesis of fibrosis.[21] miR-130 and miR301a have been reported to be activated by these cytokines in tumor and inflammatory cells. The present study found that the expression levels of miR-301a significantly increased in fibroblasts, including MEFs, HFL1 cells, and IPF fibroblasts activated with TGF-β or IL-6. Moreover, the STAT3 inhibitor S31-201 reversed miR-301a expression in fibroblasts induced by TGF-β and IL-6, thus implying that the activation of miR-301a in pulmonary fibrosis depended on STAT3. A previous study revealed that miR-301a was overexpressed during the transformation of BEAS-2B cells induced by chronic exposure to arsenic, which was controlled by the activation of STAT3 and eventually contributed to lung tumorigenesis. Increasing epidemiological evidence supports an association between IPF and lung cancer. However, the present study indicated that miR-301a might be the driving force of pulmonary fibrosis-associated lung cancer, thus suggesting promising therapeutic targets for this disease combination.

The data of the present study were distinct from previous findings in terms of increased expression levels of miR-130/301a in lung and liver tissues in pulmonary hypertension and fibrotic disease.[22] The present study demonstrated that miR-301a upregulation was profibrotic in fibroblast activation through its capacity to enhance mTOR expression. The inhibition of miR-301a has been shown to reduce mTOR activation and collagen contents using the bleomycin murine model of pulmonary fibrosis. Furthermore, the present study identified a new target of miR-301a, TSC1, in fibroblasts, and enhancing TSC1 expression in mice reduced injuries in fibrotic lung tissues.

TGF-β-induced fibroblast hyperproliferation or activation and excessive extracellular matrix deposition are the main events during fibrogenesis. The present study showed that the inhibition of miR-301a expression reduced the differentiation and proliferation of normal and IPF fibroblasts, supporting a significant correlation between miR-301a and the progression of pulmonary fibrosis. In pulmonary fibroblasts, TGF-β binds to its receptor subunits, type I and type II (TGFβR1 and TGFβR2),[23] and it activates the SMAD-signaling complex. Given that SMAD4 is an identified miR-301a target in multiple cancers, including lung,[24] prostate,[21] and pancreatic cancers,[25] the present study investigated whether SMAD4 was upregulated in fibroblasts with miR-301a inhibition. However, the expression levels of SMAD4 did not differ between these two groups, nor did the expression levels of two other miR-301a validated targets, PIAS3 and TP63, which inhibited cell proliferation. This study identified TSC1 as a novel target gene of miR-301a in fibroblasts and fibrotic mice. TSC1 is widely distributed in various organs, such as brain, lung, kidney, and heart. Increasing evidence indicates that TSC1 plays an important role in regulating cellular physiological processes, including cell proliferation and the cell

**Figure 5. Inhibition of Lung Fibrosis in *miR-301a*$^{-/-}$ Mice Correlated with Inhibition of mTOR through Upregulating TSC1 in Fibroblasts**

(A) Ki67 staining showed cell proliferation in fibrotic lung tissues from WT (n = 5) and *miR-301a*$^{-/-}$ (n = 5) mice treated with bleomycin. Scale bars, 50 μm. (B) Ki67-positive cell counts in fibrotic lung tissue sections. (C) Western blot analysis of Tsc1 expression in lung tissues from WT and *miR-301a*$^{-/-}$ mice with the indicated treatment. (D) Immunohistochemical staining for Tsc1 in fibrotic lung tissues from WT (n = 5) and *miR-301a*$^{-/-}$ (n = 5) mice treated with bleomycin. Scale bars, 50 μm. (E) Western blot analysis of phosphorylated mTOR (p-mTOR) and phosphorylated 70S6K1 (p-P70S6K1) expression in lung tissues. (F) Western blot analysis of p-mTOR and p-P70S6K1 expression in MEFs isolated from WT or *miR-301a*$^{-/-}$ mice. (G) Western blot analysis of p-mTOR and p-P70S6K1 expression in HFL1 cells transfected with either anti-Ctl or anti-miR-301a. (H) Western blot analysis of p-mTOR and p-P70S6K1 expression in IPF fibroblasts transfected with either anti-Ctl or anti-miR-301a. (I) HFL1 cells were transfected with either anti-Ctl or anti-miR-301a for 48 h and treated with TGF-β (10 ng/mL) at the indicated times. Western blot analysis of p-mTOR, p-P70S6K1, and TSC1 expression in HFL1 cells. (J) IPF fibroblasts were transfected with anti-Ctl or anti-miR-301a for 48 h and treated with TGF-β (10 ng/mL) at the indicated times. The expression of p-mTOR, p-P70S6K1, and TSC1 was measured by western blot analysis. (K) IPF fibroblasts were transfected with anti-Ctl or anti-miR-301a for 48 h and treated with IL-6 (10 ng/mL) at the indicated times. The expression of p-mTOR and TSC1 was measured by western blot analysis. (L) IPF fibroblasts were transfected with the indicated shRNA, including anti-Ctl, anti-miR-301a, or shRNA-TSC1. The expression of p-mTOR and TSC1 was measured by western blot analysis. (M) IPF fibroblasts were transfected with the indicated plasmids for 24, 48, and 72 h, respectively. Cell proliferation was measured by a CCK-8 assay. Values are expressed as mean ± standard deviation. **$p \leq 0.01$, for differences between WT and *miR-301a*$^{-/-}$ mice treated with bleomycin.

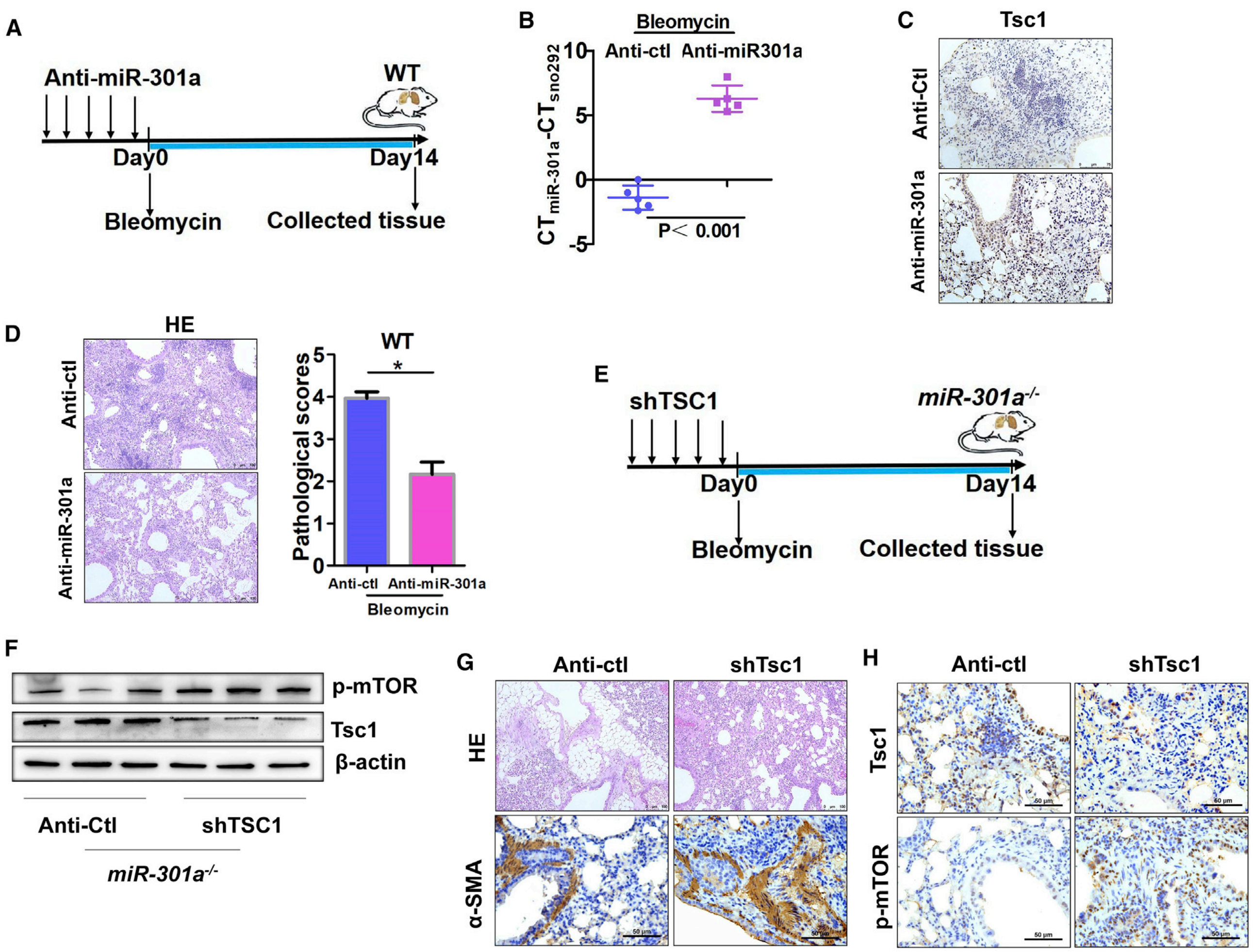


**Figure 6. Reducing Bleomycin-Induced Pulmonary Fibrosis in Mice Correlated with miR-301a Knockdown and Elevated Tsc1 Expression**

(A) Schematic representation of the tail vein injection with anti-Ctl or anti-miR-301a into WT mice, respectively (n = 5 per group). (B) The expression of miR-301a was evaluated by qPCR (n = 5 per group). (C) The expression of Tsc1 was detected by immunohistochemical analysis in WT mice. Scale bars, 75 μm. (D) H&E-stained sections and pathological scores of lung tissues. Scale bars, 100 μm. (E) Schematic representation of the tail vein injection with sh-Ctl or shTsc1 lentivirus supernatant into *miR-301a*$^{-/-}$ mice (n = 5 per group). (F) Tsc1 and p-mTOR expression in lung sections from *miR-301a*$^{-/-}$ mice (n = 3) with either sh-Ctl or shTsc1 injection was determined by western blot analysis. (G) The degree of fibrosis was shown with H&E staining (scale bars, 100 μm) and immunohistochemical analysis of α-SMA (scale bars, 50 μm) in lung tissues from *miR-301a*$^{-/-}$ mice. (H) Tsc1 and p-mTOR expression was measured by immunohistochemical staining. Scale bars, 50 μm. Values are expressed as mean ± standard deviation. **$p < 0.01$, for differences between the indicated groups.

cycle. The present study found that miR-301a negatively regulated TSC1 expression *in vitro* and *in vivo*, especially in fibroblasts. The targeted inactivation of miR-301a in fibroblasts and mouse lungs elevates the expression levels of TSC1, leading to restrained cell proliferation, extensive fibrosis, and collagen deposition in mice. Given that the miR-130/301 family members share the same "seed sequence," TSC1 expression was also upregulated by introducing the inhibitors of other miR-130/301 family members in fibroblasts, which demonstrate the extensive role of this miRNA family. Interestingly, PPARγ was only affected by miR-130a/b, but not miR-301a/b, in IPF fibroblasts, suggesting that this miRNA family may control fibrotic phenotypes *in vitro*, which are dependent on the cellular context. TSC1 is closely related to the mTOR pathway.[14] The present study next confirmed that inhibiting the expression of TSC1 in *miR-301a*$^{-/-}$ mice significantly activated mTOR and promoted pulmonary fibrosis. This study was novel in linking the inflammation stimulator miR-301a and the master regulator of cell growth and proliferation, that is, mTOR. Taken together, the present study identified a new target of miR-301a and confirmed that miR-301a activated the mTOR signaling pathway by negatively regulating TSC1, thus promoting the activation and proliferation of fibroblasts, formation of myofibroblasts, and collagen deposition and eventually leading to pulmonary fibrosis. The observations ascertained that inhibiting the miR-301a-mediated TSC1/mTOR pathway was a promising therapeutic strategy for treating fibrotic pulmonary diseases.

## MATERIALS AND METHODS

### Bleomycin Induced Pulmonary Fibrosis in Mice

The generation of *miR-301a*$^{-/-}$ mice in the C57BL/6 × 129S hybrid background has been described previously.[19] All mice were kept under pathogen-free conditions. All mouse experiments were performed and approved by the Institutional Animal Care and Use Committee of The First Affiliated Hospital of Guangzhou Medical University. Pulmonary fibrosis was induced by a single intratracheal instillation of 0.9% saline or bleomycin (3 mg/mL; Nippon Kayaku, Japan) in a volume of 50 μL, into mice. On days 14 and 21 after bleomycin instillation, the mice were sacrificed and lung tissues were rapidly collected. The tissues were fixed in 4% paraformaldehyde for histological analysis or were kept at −80°C for subsequent quantitative polymerase chain reaction (qPCR) and western blot analysis.

### Patient Data

To analyze the expression of miR-301a, lung tissues were obtained from patients with IPF. All patients were from the Department of Respiratory Medicine, The First Affiliated Hospital of Guangzhou Medical University (Table S1). All human lung tissues of IPF were obtained from biopsy using video-assisted thoracoscopic surgery following approved procedures. All diagnoses of IPF were made in accordance with the American Thoracic Society/European Respiratory Society (ATS/ERS) criteria for IPF 2011 and determined histologically. Normal donor lung tissues were obtained from mechanical pneumothorax patients. The study protocol was approved by the Clinical Research Ethics Committee of the Guangzhou Medical University. Written informed consent was obtained from all participants before starting the study.

### Cell Culture and Reagents

The HFL1 cell line was purchased and authenticated from the Cell Bank of the Chinese Academy of Sciences (Shanghai, China). Based on previous experiments, human fibroblasts were extracted from patients diagnosed with IPF using lung biopsies.[26] All cells were cultured in Dulbecco's modified Eagle's medium (DMEM)/nutrient mixture F-12 supplemented with 10% fetal bovine serum (FBS). MEF fibroblasts were isolated from the embryos of WT and *miR-301a*$^{-/-}$ mice and cultured in MEF medium (DMEM supplemented with 10% FBS, 1% penicillin/streptomycin, and 1% non-essential amino acids [NEAAs]). All cell lines were cultured at 37°C with 5% $CO_2$. Human recombinant TGF-β and IL-6 were purchased from PeproTech (Shuzhou, China). LNA-anti-Control and LNA-anti-miR-301a for *in vitro* applications were purchased from Exiqon (MA, USA).

### Cell Proliferation Assay

The cells were seeded in a 96-well plate, transfected with LNA-anti-control or LNA-anti-miR-301a, and then treated with TGF-β (10 ng/mL) to evaluate cell viability at different time points. Cell proliferation was determined using Cell Counting Kit-8 (CCK-8; Dojindo Molecular Technologies, China) following the manufacturer's protocol.

### Cell Transfection

HFL1 cells and IPF fibroblasts were transfected with LNA-anti-control or LNA-anti-miR-301a using Lipofectamine RNAiMAX reagent. For the knockdown of TSC1 in human cells, the shTSC1-1 (5′-GCACTCTTTCATCGCCTTTAT-3′), shTSC1-2 (5′-CACCTCTTG GACAGGATTAAC-3′), shTSC1-3 (5′-AAAGAAGAAGCTGCAA TATCT-3′), shTSC1-4 (5′-GCAGCCATCTTGGAAGCATAA-3′), and nonsense sequence (shRNA-control, 5′-GGTGTGCAGTTG GAATGTA-3′) were synthesized and ligated into an miRZip lentivector-based anti-miRNA vector (System Biosciences, USA). TSC1 cDNA was amplified and ligated into the same plasmid. HEK293T cells were seeded in a six-well plate and transfected with 0.75 μg of pRSV-rev, 0.75 μg of pMDL-rre, 0.75 μg of pMD2.G, and 1 μg of shRNA plasmid construct. The cells were cultured at 37°C for 48 h, and the supernatant with the virus was harvested. Then, the fibroblasts were infected with the virus in the presence of 8 μg/mL Polybrene; the medium was changed 6 h after infection.

### Quantitative Real-Time PCR

Total RNA was isolated using TRIzol (Invitrogen, USA) following the manufacturer's recommendations. The RNA was reverse transcribed with an iScript reverse transcription supermix kit (Bio-Rad, CA, USA) following the manufacturer's protocol. Real-time PCR for miR-301a detection was performed using the TaqMan assay (Thermo Fisher Scientific, USA) with sno292 (mice) or U6 (human) small nuclear RNA as a reference and a CFX96 real-time PCR detection system (Bio-Rad, USA).

### Western Blot Analysis

The proteins were extracted from fibroblasts or lung tissues using radioimmunoprecipitation assay (RIPA) lysis buffer (Cell Signaling Technology, USA) supplemented with a protease inhibitor (Thermo Fisher Scientific, USA). The proteins were then subjected to sodium dodecyl sulfate-polyacrylamide gel electrophoresis, transferred onto polyvinylidene fluoride membranes (Immobilon-P transfer membrane), blocked with 5% nonfat dry milk for 1 h, and then incubated with the primary antibodies at 4°C overnight. The primary antibodies were as follows: TSC1 (20988-1-1AP, Proteintech, USA), p-mTOR-S2448 (381557, ZenBio, USA), p-P70S6K1-T389 (AP0564, ABclonal, China), anti-Fn (ab2413, Abcam, USA), anti-α-SMA (ab5694, Abcam), vimentin (A11952, ABclonal), Ki67 (A2094, ABclonal), STAT3 (9139, CST, USA), p-Stat3-Tyr705 (9145, CST), PTEN (A11193, ABclonal), SMAD4 (38454, CST), TP63 (A12968, ABclonal), PIAS3 (A7060, ABclonal), and β-actin (AC026, ABclonal). After washing with Tris-buffered saline (TBS) with Tween 20 (TBST) buffer, the membranes were incubated for 1 h at room temperature with a horseradish peroxidase (HRP)-conjugated anti-rabbit or anti-mouse secondary antibody. The immunoreactive proteins were visualized with a SuperSignal West Pico chemiluminescent substrate (Thermo Fisher Scientific, USA). The images were scanned using a Tanon 5200 automatic chemiluminescence imaging analysis system and acquired using PVCAM software (Tanon, China). All experiments were performed in triplicate.

### Immunohistochemistry

The lung tissues were fixed in 10% formalin solution. Paraffin was used to encapsulate the chips, and then 3-μm-thick slices were prepared. The sections were stained with H&E and Masson's trichrome to detect interstitial volume expansion. The antigen was repaired with sodium citrate buffer in a water bath at 96°C. After washing with phosphate-buffered saline three times and 3% $H_2O_2$ for 5 min, the sections were blocked with 10% normal goat serum. For immunohistochemical staining to measure the levels of α-SMA, Ki67, Tsc1, and p-mTOR, the lung tissue sections were incubated with these primary antibodies at 4°C overnight. The tissue sections were developed using an Ultra Vision Detection System (Thermo Fisher Scientific, MA, USA).

### Luciferase Reporter Assay

The 3′ UTR sequence of TSC1, including two predicted miRNA recognition elements, was synthesized and subcloned into the psiCHECK-2 (C8021, Promega, USA) dual luciferase reporter vector. HEK293T cells were transfected with the psiCHECK-2 vector using TSC1 3′ UTR or pre-miR-301a or pre-miR-control. Then, cells were lysed and the activities of firefly and Renilla luciferases were determined using a dual luciferase reporter assay system (GeneCopoeia, USA) 48 h after transfection following the manufacturer's protocol. Normalized data were calculated as the ratio of Renilla/firefly luciferase activities.

### *In Vivo* Administration of LNA-Anti-miR-301a or Tsc1 shRNA Lentivirus

For miR-301a inhibition in murine fibrosis, LNA-modified antisense to miR-301a or LNA miRNA inhibitor control was intraperitoneally injected into mice; the total dose for anti-miR-301a or control-anti-miR was 20 mg/kg of body weight. For the knockdown of Tsc1 in mice, shRNA-Tsc1 (5′-CAACACGTTGGTTGATTATTA-3′) or Tsc1 sequence with nonsense mutation (shRNA-control, 5′-GTGTGCAGTTGGAATGTA-3′) was synthesized and ligated into miRZip lentivector-based vectors. The lentivirus was prepared in HEK293 T cells, and then the supernatant containing the virus ($1 \times 10^8$ plaque-forming units [PFU]) was injected into mice via the tail vein. After 5 days of continuous injection with the lentivirus, the mice were intratracheally instilled with bleomycin, and the lung tissues were collected on day 14. Western blot analysis was performed to ensure that the silencing was achieved.

### Statistical Analysis

Data were expressed as the mean ± standard deviation of at least three independent experiments. The results were statistically analyzed using a two-tailed Student t test for two-group comparisons or one-way analysis of variance for multiple-group comparisons. The statistical significance was set at a p value <0.05 or a p value <0.01.

## SUPPLEMENTAL INFORMATION

Supplemental Information can be found online at https://doi.org/10.1016/j.omtn.2020.05.027.

## AUTHOR CONTRIBUTIONS

J.W., X.L., and M.Z. performed the experiments. L.Z. and Y.W. participated in data analysis and animal experiments. M.W., X.G., and X.W. assisted in immunostaining, western blot analysis, and data interpretation. C.Z., M.L., and X.M. designed and wrote the manuscript. All authors reviewed the manuscript and approved the submission.

## CONFLICTS OF INTEREST

The authors declare no competing interests.

## ACKNOWLEDGMENTS

This work was supported by grants from the National Natural Science Foundation of China (81773249 and 81572399), the Natural Science Foundation of Guangdong Province of China (2017A030311004), the Science and Technology Program of Guangzhou City of China (201707010139), and by a grant to X.L. from the South China Normal University Graduate Student Innovation Plan Project (2019LKXM002).

## REFERENCES

1. Stowasser, S., and Hallmann, C. (2015). New guideline for idiopathic pulmonary fibrosis. Lancet *386*, 1823–1824.
2. Vancheri, C., and du Bois, R.M. (2013). A progression-free end-point for idiopathic pulmonary fibrosis trials: lessons from cancer. Eur. Respir. J. *41*, 262–269.
3. Noble, P.W., Albera, C., Bradford, W.Z., Costabel, U., Glassberg, M.K., Kardatzke, D., King, T.E., Jr., Lancaster, L., Sahn, S.A., Szwarcberg, J., et al.; CAPACITY Study Group (2011). Pirfenidone in patients with idiopathic pulmonary fibrosis (CAPACITY): two randomised trials. Lancet *377*, 1760–1769.
4. Distler, O., Highland, K.B., Gahlemann, M., Azuma, A., Fischer, A., Mayes, M.D., Raghu, G., Sauter, W., Girard, M., Alves, M., et al.; SENSCIS Trial Investigators (2019). Nintedanib for systemic sclerosis-associated interstitial lung disease. N. Engl. J. Med. *380*, 2518–2528.
5. Sahin, H., and Wasmuth, H.E. (2013). Chemokines in tissue fibrosis. Biochim. Biophys. Acta *1832*, 1041–1048.
6. Shukla, G.C., Singh, J., and Barik, S. (2011). MicroRNAs: processing, maturation, target recognition and regulatory functions. Mol. Cell. Pharmacol. *3*, 83–92.
7. Asanoma, K., Hori, E., Yoshida, S., Yagi, H., Onoyama, I., Kodama, K., Yasunaga, M., Ohgami, T., Kaneki, E., Okugawa, K., et al. (2019). Mutual suppression between BHLHE40/BHLHE41 and the MIR301B-MIR130B cluster is involved in epithelial-to-mesenchymal transition of endometrial cancer cells. Oncotarget *10*, 4640–4654.
8. Bertero, T., Cottrill, K., Krauszman, A., Lu, Y., Annis, S., Hale, A., Bhat, B., Waxman, A.B., Chau, B.N., Kuebler, W.M., and Chan, S.Y. (2015). The microRNA-130/301 family controls vasoconstriction in pulmonary hypertension. J. Biol. Chem. *290*, 2069–2085.
9. Ohsawa, M., Kobayashi, T., Okura, H., Igarashi, T., Mizuguchi, M., and Hino, O. (2013). TSC1 controls distribution of actin fibers through its effect on function of Rho family of small GTPases and regulates cell migration and polarity. PLoS ONE *8*, e54503.
10. Marcotte, L., and Crino, P.B. (2006). The neurobiology of the tuberous sclerosis complex. Neuromolecular Med. *8*, 531–546.
11. Fingar, D.C., Richardson, C.J., Tee, A.R., Cheatham, L., Tsou, C., and Blenis, J. (2004). mTOR controls cell cycle progression through its cell growth effectors S6K1 and 4E-BP1/eukaryotic translation initiation factor 4E. Mol. Cell. Biol. *24*, 200–216.
12. Gui, Y.S., Wang, L., Tian, X., Li, X., Ma, A., Zhou, W., Zeng, N., Zhang, J., Cai, B., Zhang, H., et al. (2015). mTOR overactivation and compromised autophagy in the pathogenesis of pulmonary fibrosis. PLoS ONE *10*, e0138625.
13. Han, Q., Lin, L., Zhao, B., Wang, N., and Liu, X. (2018). Inhibition of mTOR ameliorates bleomycin-induced pulmonary fibrosis by regulating epithelial-mesenchymal transition. Biochem. Biophys. Res. Commun. *500*, 839–845.

14. Woodcock, H.V., Eley, J.D., Guillotin, D., Platé, M., Nanthakumar, C.B., Martufi, M., Peace, S., Joberty, G., Poeckel, D., Good, R.B., et al. (2019). The mTORC1/4E-BP1 axis represents a critical signaling node during fibrogenesis. Nat. Commun. *10*, 6.

15. Lawrence, J., and Nho, R. (2018). The role of the mammalian target of rapamycin (mTOR) in pulmonary fibrosis. Int. J. Mol. Sci. *19*, 778.

16. Wang, Y., Chen, C., Deng, Z., Bian, E., Huang, C., Lei, T., Lv, X., Liu, L., and Li, J. (2017). Repression of TSC1/TSC2 mediated by MeCP2 regulates human embryo lung fibroblast cell differentiation and proliferation. Int. J. Biol. Macromol. *96*, 578–588.

17. Holm Nielsen, S., Willumsen, N., Leeming, D.J., Daniels, S.J., Brix, S., Karsdal, M.A., Genovese, F., and Nielsen, M.J. (2019). Serological assessment of activated fibroblasts by alpha-smooth muscle actin (α-SMA): a noninvasive biomarker of activated fibroblasts in lung disorders. Transl. Oncol. *12*, 368–374.

18. Mycko, M.P., Cichalewska, M., Machlanska, A., Cwiklinska, H., Mariasiewicz, M., and Selmaj, K.W. (2012). MicroRNA-301a regulation of a T-helper 17 immune response controls autoimmune demyelination. Proc. Natl. Acad. Sci. USA *109*, E1248–E1257.

19. Ma, X., Yan, F., Deng, Q., Li, F., Lu, Z., Liu, M., Wang, L., Conklin, D.J., McCracken, J., Srivastava, S., et al. (2015). Modulation of tumorigenesis by the pro-inflammatory microRNA miR-301a in mouse models of lung cancer and colorectal cancer. Cell Discov. *1*, 15005.

20. Li, X., Zhong, M., Wang, J., Wang, L., Lin, Z., Cao, Z., Huang, Z., Zhang, F., Li, Y., Liu, M., and Ma, X. (2019). miR-301a promotes lung tumorigenesis by suppressing Runx3. Mol. Cancer *18*, 99.

21. Li, X., Li, J., Cai, Y., Peng, S., Wang, J., Xiao, Z., Wang, Y., Tao, Y., Li, J., Leng, Q., et al. (2018). Hyperglycaemia-induced miR-301a promotes cell proliferation by repressing p21 and Smad4 in prostate cancer. Cancer Lett. *418*, 211–220.

22. Bertero, T., Cottrill, K.A., Annis, S., Bhat, B., Gochuico, B.R., Osorio, J.C., Rosas, I., Haley, K.J., Corey, K.E., Chung, R.T., et al. (2015). A YAP/TAZ-miR-130/301 molecular circuit exerts systems-level control of fibrosis in a network of human diseases and physiologic conditions. Sci. Rep. *5*, 18277.

23. Thomas, B.J., Kan-O, K., Loveland, K.L., Elias, J.A., and Bardin, P.G. (2016). In the shadow of fibrosis: innate immune suppression mediated by transforming growth factor-β. Am. J. Respir. Cell Mol. Biol. *55*, 759–766.

24. Zhong, M., Huang, Z., Wang, L., Lin, Z., Cao, Z., Li, X., Zhang, F., Wang, H., Li, Y., and Ma, X. (2018). Malignant transformation of human bronchial epithelial cells induced by arsenic through STAT3/miR-301a/SMAD4 loop. Sci. Rep. *8*, 13291.

25. Xia, X., Zhang, K., Cen, G., Jiang, T., Cao, J., Huang, K., Huang, C., Zhao, Q., and Qiu, Z. (2015). MicroRNA-301a-3p promotes pancreatic cancer progression via negative regulation of SMAD4. Oncotarget *6*, 21046–21063.

26. Liu, M., Zeng, X., Wang, J., Fu, Z., Wang, J., Liu, M., Ren, D., Yu, B., Zheng, L., Hu, X., et al. (2016). Immunomodulation by mesenchymal stem cells in treating human autoimmune disease-associated lung fibrosis. Stem Cell Res. Ther. *7*, 63.

OMTN, Volume *21*

## Supplemental Information

# miR-301a Suppression within Fibroblasts
# Limits the Progression of Fibrosis
# through the TSC1/mTOR Pathway

**Jiexuan Wang, Xun Li, Mingtian Zhong, Yansheng Wang, Liming Zou, Miaomiao Wang, Xiaoli Gong, Xinjie Wang, Chengzhi Zhou, Xiaodong Ma, and Ming Liu**

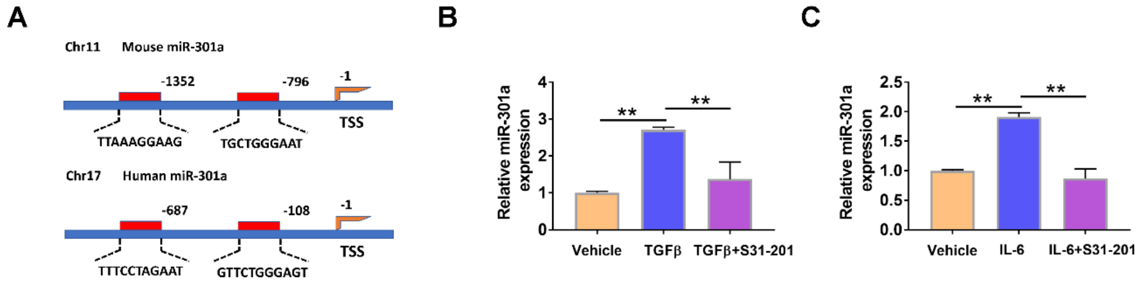


**Supplementary Figure 1. miR-301a expression in fibroblast activation is dependent on Stat3.** **(A)** Online prediction indicated that transcription factor Stat3 might bind to the miR-301a promoter in both mouse and human. Fibroblasts were treated with TGF-β **(B)** or IL-6 **(C)** and Stat3 inhibitor, S31-201 (30μM) at 24 h and the expression of miR-301a was determined by qPCR. Values are means ± sd. ***P* ≤0.01 indicate the significance of the differences between the indicated groups.

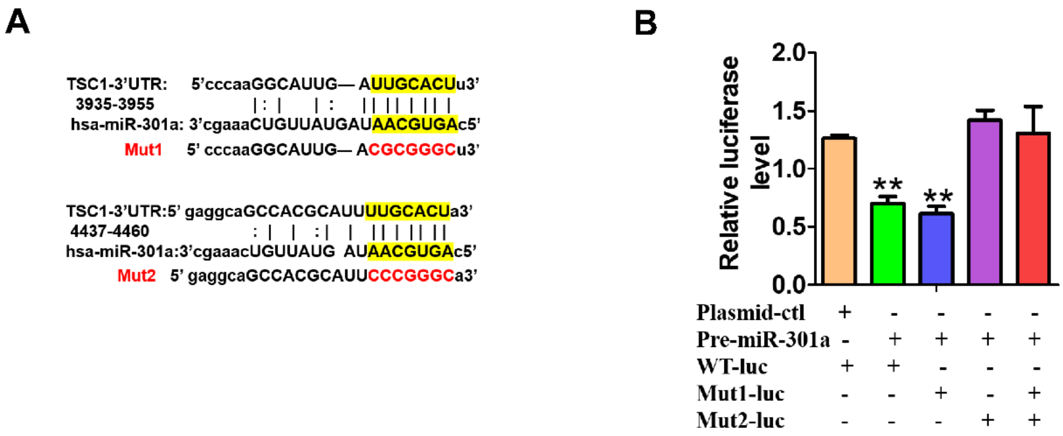


**Supplementary Figure 2. The true binding site of miR-301a with TSC1. (A)** Prediction of major interference sites between miR-301a and TSC1 mRNA 3'UTR by using Targetscan. Mutant 3′UTR contains mutations that abolished the seed match with miR-301a. **(B)** Luciferase activity in 293T cells transfected with the indicated luciferase reporters.

**A**

miR-130a CAGUGCAAUGUUAAAAGGGCAU
miR-130b CAGUGCAAUGAUGAAAGGGCAU
miR-301a CAGUGCAAUAGUAUUGUCAAAGC
miR-301b CAGUGCAAUGAUAUUGUCAAAGC

**B**

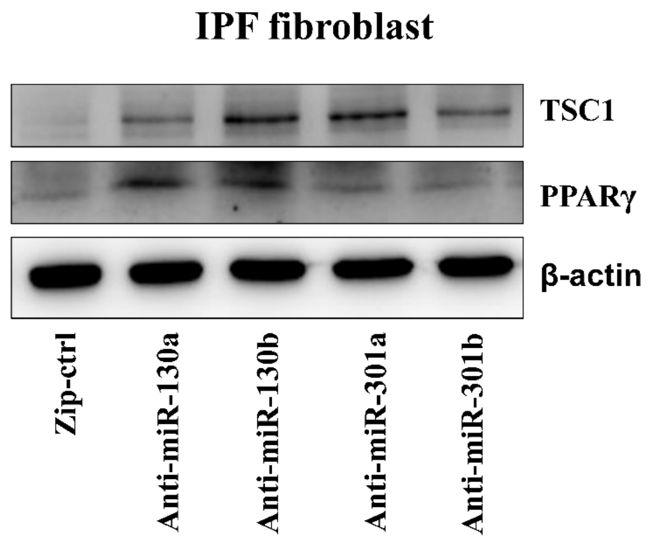


**Supplementary Figure 3. (A)** Prediction of major interference sites between miR-301a and TSC1 mRNA 3'UTR by using Targetscan. **(B)** Luciferase activity in 293T cells transfected with the indicated luciferase reporter with either a control plasmid or precursor miR-301a plasmid.

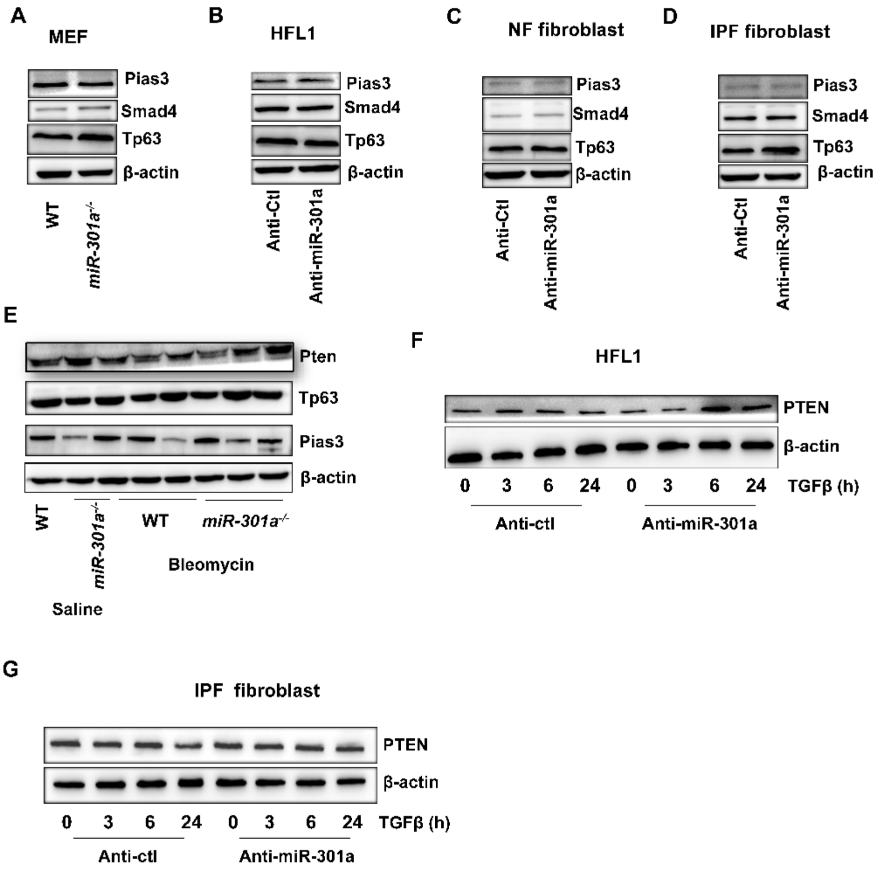


**Supplementary Figure 4. Expression of miR-301a targets in fibroblast cells. (A)** Western blot analysis of the expression of miR-301a targets Pias3, Smad4, and Tp63 in WT and *miR-301a*$^{-/-}$ MEFs. **(B–D)** Western blot analysis of the expression of miR-301a targets Pias3, Smad4, and Tp63 in HFL1 cells **(B)**, NF fibroblasts isolated from a normal donor **(C)**, and fibroblasts from patients with IPF **(D)**, which were transfected with either Anti-Ctl or Anti-miR-301a. **(E)** Western blot analysis of the expression of miR-301a targets PTEN, Tp63, and Pias3 in lung tissues from WT and *miR-301a*$^{-/-}$ mice with the indicated treatment. Western blot analysis of PTEN expression in HFL1 cells **(F)** and IPF fibroblasts **(G)**, which were transfected with either Anti-Ctl or Anti-miR-301a.

A

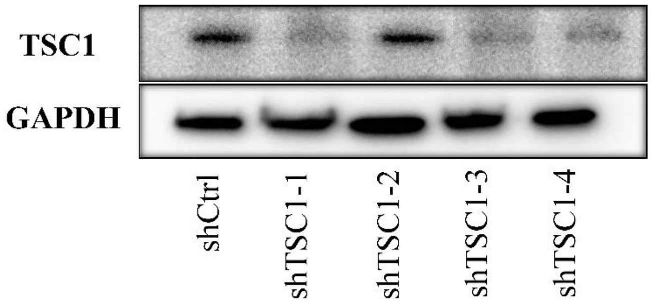


**Supplementary Figure 5. Western blot shows the efficacy of shRNA knockdown of TSC1 in 293T cells.**

**Supplementary Table 1. The characteristics of IPF patient.**

| Characteristics | Range（N=8） |
|---|---|
| Age | 54.75（36-64） |
| Sex | |
| Male | 3 |
| Female | 5 |
| Pathologic diagnosis | UIP |
| Therapy | |
| Prednisone | 8 |
| N-Acetyl-L-cysteine | 8 |
| Nintedanib | 2 |
| Cyclophosphamide(CTX) | 1 |
| Azathioprine | 1 |
